# Agomelatine Changed the Expression and Methylation Status of Inflammatory Genes in Blood and Brain Structures of Male Wistar Rats after Chronic Mild Stress Procedure

**DOI:** 10.3390/ijms23168983

**Published:** 2022-08-11

**Authors:** Katarzyna Bialek, Piotr Czarny, Paulina Wigner, Ewelina Synowiec, Lukasz Kolodziej, Michal Bijak, Janusz Szemraj, Mariusz Papp, Tomasz Sliwinski

**Affiliations:** 1 Department of Medical Biochemistry, Medical University of Lodz, 92-215 Lodz, Poland; 2 Department of General Biochemistry, Faculty of Biology and Environmental Protection, University of Lodz, 90-236 Lodz, Poland; 3 Laboratory of Medical Genetics, Faculty of Biology and Environmental Protection, University of Lodz, 90-236 Lodz, Poland; 4 Biohazard Prevention Centre, Faculty of Biology and Environmental Protection, University of Lodz, 90-236 Lodz, Poland; 5 Institute of Pharmacology, Polish Academy of Sciences, 31-343 Krakow, Poland

**Keywords:** depression, chronic mild stress, agomelatine, inflammation, expression, methylation

## Abstract

The preclinical research conducted so far suggest that depression development may be influenced by the inflammatory pathways both at the periphery and within the central nervous system. Furthermore, inflammation is considered to be strongly connected with antidepressant treatment resistance. Thus, this study explores whether the chronic mild stress (CMS) procedure and agomelatine treatment induce changes in *TGFA*, *TGFB*, *IRF1*, *PTGS2* and *IKBKB* expression and methylation status in peripheral blood mononuclear cells (PBMCs) and in the brain structures of rats. Adult male Wistar rats were subjected to the CMS and further divided into matched subgroups to receive vehicle or agomelatine. TaqMan gene expression assay and methylation-sensitive high-resolution melting (MS-HRM) were used to evaluate the expression of the genes and the methylation status of their promoters, respectively. Our findings confirm that both CMS and antidepressant agomelatine treatment influenced the expression level and methylation status of the promoter region of investigated genes in PBMCs and the brain. What is more, the present study showed that response to either stress stimuli or agomelatine differed between brain structures. Concluding, our results indicate that *TGFA*, *TGFB*, *PTGS2*, *IRF1* and *IKBKB* could be associated with depression and its treatment.

## 1. Introduction

Clinical studies showed that depression (major depressive disorder, MDD), being one of the most frequently diagnosed mental diseases, affects more than 260 million people worldwide. This severe psychiatric condition has a lifetime prevalence in excess of 15% and is a significant contributor to the global burden of disease [1]. MDD is estimated to be the second leading cause of disability and morbidity. Complex symptoms, often chronic or recurrent, limit people’s functioning, affect their mental, social, physical well-being and may lead to suicide [2]. Furthermore, one of the major problems associated with depression is the high rate of relapse as well as the fact that more than one-third of patients do not respond to antidepressant treatment [3,4]. On these bases, it is important to identify the pathogenesis and molecular mechanisms involved in depression.

Among the systems contributing to disease development, there is a growing body of evidence suggesting that it may be influenced by the activation of the immune system and thus the elevated expression and activity of pro-inflammatory agents [5]. A great amount of various experimental evidence indicates the derangement and increased concentration of cytokines as well as other inflammatory markers including acute phase reactants, prostaglandins, adhesion molecules and chemokines in MDD patients [6,7,8,9]. Indeed, inflammation is an important hallmark of MDD. Moreover, these molecules act as neuromodulators and can trigger a cascade of neurochemical, neuroendocrine and behavioral changes [10]. Importantly, peripheral inflammation is reflected in the central nervous system (CNS), and it is characterized by the activation of immune cells called microglia [11]. Moreover, microglia mobilization is mostly correlated with the activation of nuclear factor-kB (NF-kB) responsible for cytokine production [12]. Furthermore, NF-kB regulates neurogenesis and synaptic plasticity in the nervous system by interacting with the brain-derived neurotrophic factor (BDNF), which is a cornerstone of the neurotrophic hypothesis of depression [13,14]. This could be a major contributor to neuroinflammation, disrupting the neurotrophic system and affecting regional brain activity [15]. Inflammation is known to be especially abundant in areas of the brain such as the frontal cortex, hypothalamus, and hippocampus that are particularly sensitive to chronic stress [16,17]. What is more, it is hypothesized that some antidepressant drugs have been shown to effectively diminish pro-inflammatory cytokines levels [18]. Moreover, evidence also suggest that some cytokines may play an important role in the response to the antidepressant treatment [19]. More specifically, these findings are even more significant considering that elevated levels of specific inflammatory mediators are strongly related with treatment-resistant depression [20,21]. Currently, selective serotonin reuptake inhibitors (SSRIs) and serotonin–norepinephrine reuptake inhibitors (SNRIs), tricyclic antidepressants (TCAs) and monoamine oxidase inhibitors (MAOIs) are used in the first-line treatment of MDD [22,23]. However, antidepressant agents administration is often associated with adverse effects and incomplete tolerability in depressed subjects. Moreover, there are also many limitations of current therapeutic approaches; for example, patients often fail to respond to the treatment or experience recurrence, which is possibly due to a number of biological systems affected in depression [24,25].

The relatively novel antidepressant drug, agomelatine, has been considered to have advantages over the other drugs [26]. It acts as an potent agonist of MT1 and MT2 melatonin receptors, with additional antagonist properties at the serotonin 5-HT_2c_ receptor subtype [26,27]. There is evidence that agomelatine is associated with enhanced neurogenesis and an increased expression of *BDNF* [28]. Moreover, it has been shown that agomelatine has some anti-inflammatory properties on the periphery, and it is also able to ameliorate the neuroinflammatory processes [29]. However, the chronic impact of agomelatine administration on the levels of inflammatory molecules, other than cytokines, in the peripheral and central nervous system has been barely studied.

This study focuses on the contribution of genes such as *IRF1, PTGS2, IKBKB, TGFA* and *TGFB* in the molecular basis of depression as well as the impact of chronic agomelatine administration. *TGFA* and *TGFB* encode for polypeptide growth factors, Transforming Growth Factor Alpha and Transforming Growth Factor Beta; one of their main functions is regulation of the immune system [30,31]. Moreover, *TGFB* is known to play a neuroprotective role, especially in neurodegenerative disorders [32] as well as stimulate prostaglandin-endoperoxide synthase 2 (PTGS2; cyclooxygenase-2—COX-2) encoded by the *PTGS2* gene, which is associated with depression etiology and progression [33,34]. Another molecule involved in inflammatory pathways is interferon regulatory factor 1 (IRF1), which is a transcriptional factor for many genes associated with inflammation [35]. What is more, IRF1 interacts with NF-kB, which is implicated in MDD pathogenesis. NF-kB is also responsible for the regulation of neurogenesis and maintaining plasticity in the nervous system [36,37]. Canonical NF-kB signaling is regulated by the IkB kinase, consisting of three units. One of these subunits is IKK-B (inhibitor of nuclear factor kappa-B kinase subunit), which is encoded by the *IKBKB* gene [38,39,40]. Thus, the dysregulation of *IKBKB* gene expression might cause disturbances in the NF-kB system and influence the development of MDD. Taken together, these genes and their protein products are known to play a role in inflammatory processes in the periphery and CNS; thus, they could be implicated in MDD pathogenesis. Importantly, as it is only possible to directly study the brain of depressed patients post mortem, such research requires the use of an animal model, including chronic mild stress (CMS), to understand the complex relationship between many processes, including inflammation.

Therefore, the purpose of the present study was to explore whether: (1) the CMS procedure in rats, which closely mirrors depression in humans, induces changes in *TGFA, TGFB, IRF1, PTGS2* and *IKBKB* expression at the mRNA level in peripheral blood mononuclear cells (PBMCs) and in selected brain structures (hippocampus, amygdala, midbrain, hypothalamus, prefrontal cortex and basal ganglia); (2) the chronic administration of agomelatine alters the expression of these genes in the peripheral and central nervous system; (3) the CMS procedure and chronic agomelatine administration cause epigenetic changes in the investigated genes such as the methylation level in their promoters.

## 2. Results

### 2.1. Sucrose Intakes and Body Weights of Animals Exposed to CMS and Agomelatine Administration

The 1% sucrose solution consumption was comparable in all groups before the CMS procedure was initiated (Week 0). After the two-week CMS procedure, sucrose consumption decreased to approximately 50% of initial values in all stressed groups (Week 2; Stressed, Stressed/Saline, Stressed/Agomelatine). Intakes remained at low levels in stressed animals administered with vehicle until the end of the experiment (Week 7; Stressed/Saline). Although chronic (five-week) agomelatine treatment yielded no effect in control animals, it normalized the sucrose intake in stressed rats (Table 1). Both stress and agomelatine had no significant effect on the body weights of the control or CMS animals.

### 2.2. Gene Expression

#### 2.2.1. Gene Expression in PBMCs after CMS Procedure and Agomelatine Administration

**Figure 1:**
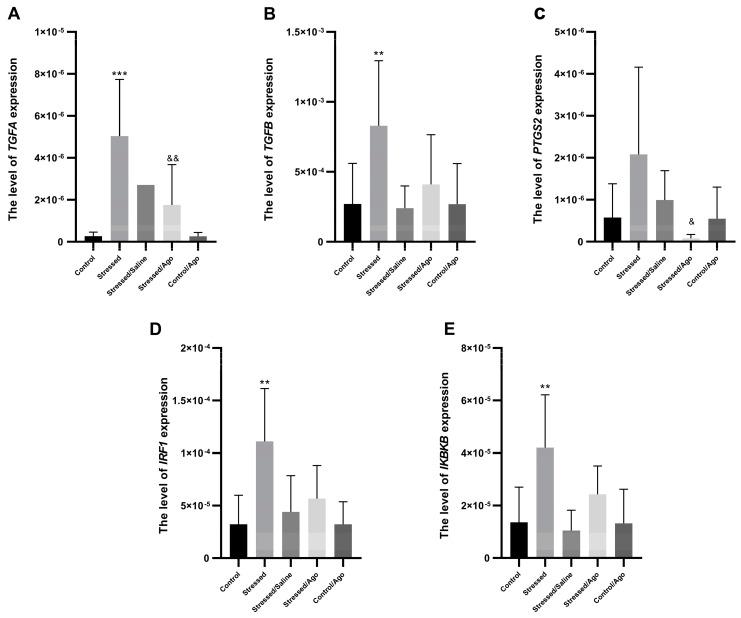
mRNA expression of *TGFA* (**A**), *TGFB* (**B**), *PTGS2* (**C**), *IRF1* (**D**) and *IKBKB* (**E**) in PBMCs of animals exposed to chronic mild stress (CMS) for two weeks (Control, Stressed) and in animals exposed to CMS for seven weeks and administered vehicle (1 mL/kg) or venlafaxine (10 mg/kg) for five weeks (Stressed/Saline, Stressed/Ago, Control/Ago). Relative gene expression levels were estimated using the 2−ΔCt (Ctgene–Ct18S) method. Data represent means ± SD. *N* = 6; ** *p* < 0.01; *** *p* < 0.001 relative to Control group; & *p* < 0.05, && *p* < 0.01 relative to Stressed group. *TGFA* (**A**): Stressed vs. Control ANOVA *p* < 0.001, Tukey’s test *p* < 0.001; Stressed/Ago vs. Stressed ANOVA *p* < 0.001, Tukey’s test *p* = 0.006; *TGFB* (**B**): Stressed vs Control ANOVA *p* = 0.002, Tukey’s test *p* = 0.004; *PTGS2*(**C**): Stressed/Ago vs. Stressed ANOVA *p* = 0.045, Tukey’s test *p* = 0.031. *IRF1* (**D**): Stressed vs Control ANOVA *p* = 0.003, Tukey’s test *p* = 0.005. *IKBKB* (**E**): Stressed vs. Control ANOVA *p* = 0.003, Tukey’s test *p* = 0.01.

The chronic mild stress procedure for the initial 2 weeks caused significant changes in the mRNA expression level of *TGFA, TGFB, IRF1* and *IKBKB* in PBMCs (Figure 1). Stressed animals demonstrated increased mRNA levels of these genes compared to the control group. The chronic treatment of agomelatine for five weeks yielded no effect relative to placebo administration. However, in the case of *TGFA* and *PTGS2,* a significant expression decrease could be observed in the agomelatine group compared to the CMS group.

#### 2.2.2. Gene Expression in the Brain after CMS Procedure and Agomelatine Administration

**Figure 2:**
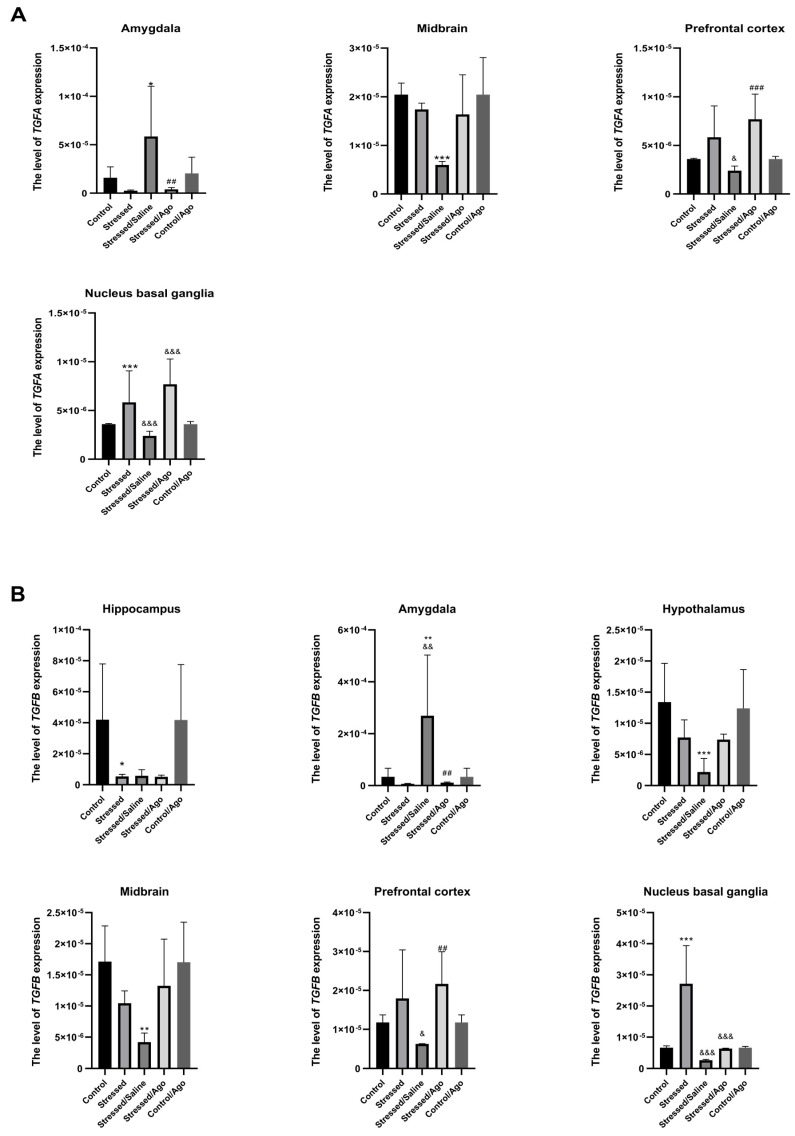

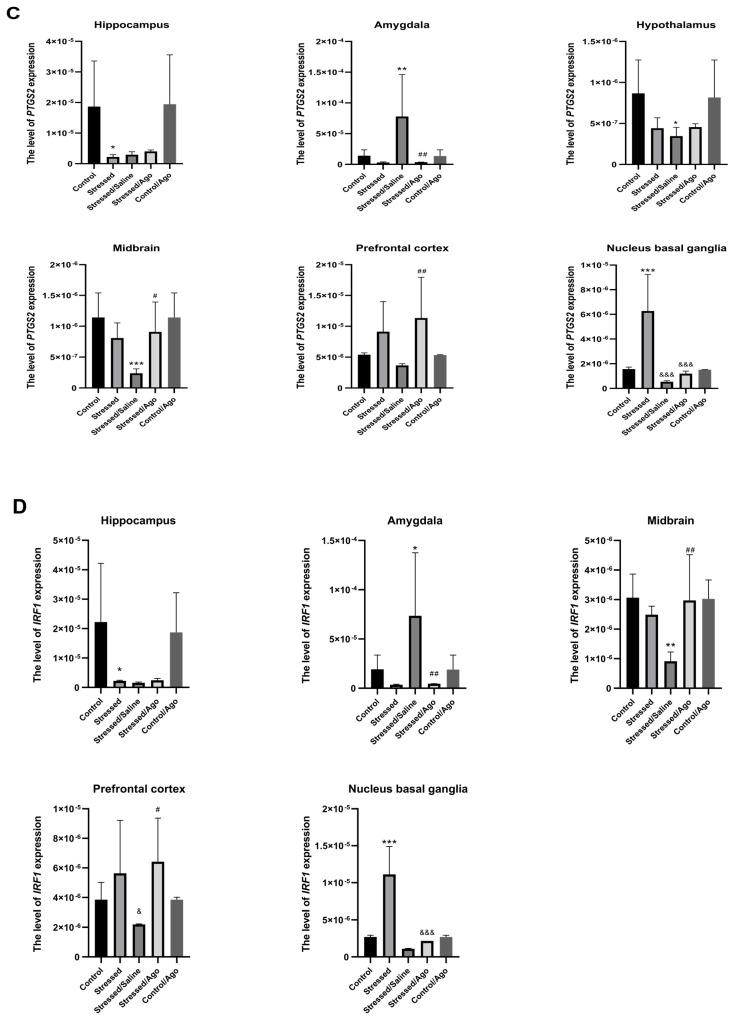

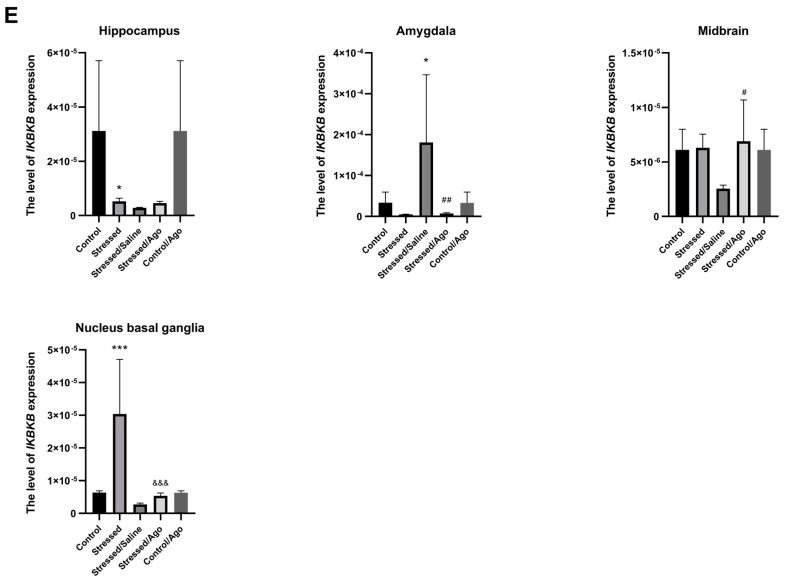
mRNA expression of TGFA (**A**), IKBKB (**B**), TGFB (**C**), IRF1 (**D**) and PTGS2 (**E**) in the brain structures of animals exposed to chronic mild stress (CMS) for two weeks (Control, Stressed) and in animals exposed to CMS for seven weeks and administered vehicle (1 mL/kg) or venlafaxine (10 mg/kg) for five weeks (Control/Venla, Stressed/Saline, Stressed/Venla). Relative gene expression levels were estimated using a 2−ΔCt (Ctgene–Ct18S) method. Data represent means ± SD. *N* = 6. * *p* < 0.05; ** *p* < 0.01; *** *p* < 0.001 relative to Control group. # *p* < 0.05; ## *p* < 0.01; ### *p* < 0.001 relative to Stressed/Saline group. & *p* < 0.05; && *p* < 0.01; &&& *p* < 0.001 relative to Stressed group.

The effect of CMS procedure and antidepressant treatment on the mRNA expression level of studied genes differed between structures of the brain (Figure 2A–E). CMS for the initial two weeks caused a significant up-regulation of *TGFA, TGFB, PTGS2, IRF1* and *IKBKB* expression in the nucleus basal ganglia. Simultaneously, it induced the down-regulation of *TGFB, PTGS2, IRF1* and *IKBKB* in the hippocampus. Furthermore, prolonged exposure to stress for seven weeks in the placebo group led to an increase in the expression of all studied genes in the amygdala. Interestingly, animals after the seven-week CMS demonstrated decreased mRNA levels of *TGFA, TGFB, PTGS2,* and *IRF1* in the midbrain and *TGFB* and *PTGS2* in the hypothalamus. Chronic agomelatine administration for five weeks induced an up-regulation of *TGFA, TGFB, PTGS2, IRF1* and *IKBKB* expression in the prefrontal cortex as well as *PTGS2* and *IRF1* in the midbrain. On the other hand, agomelatine treatment also decreased the expression of *TGFA, TGFB, PTGS2, IRF1* and *IKBKB* in the amygdala.

*TGFA (A)* Amygdala Stressed/Saline vs. Control ANOVA *p* = 0.004, Tukey’s test *p* = 0.046; Stressed/Ago vs. Stressed/Saline ANOVA *p* = 0.004, Tukey’s test *p* = 0.007; Midbrain Stressed/Saline vs. Control ANOVA *p* < 0.001, Tukey’s test *p* < 0.001; Prefrontal cortex Stressed/Ago vs. Stressed/Saline ANOVA *p* < 0.001, Tukey’s test *p* < 0.001; Nucleus basal ganglia Stressed vs. Control ANOVA *p* < 0.001, Tukey’s test *p* < 0.001, Stressed/Ago vs. Stressed ANOVA *p* < 0.001, Tukey’s test *p* < 0.001.

*TGFB (B)* Hippocampus Stressed vs. Control ANOVA *p* = 0.006, Tukey’s test *p* < 0.067; Amygdala Stressed/saline vs. Control ANOVA *p* < 0.001, Tukey’s test *p* = 0.007, Stressed/Ago vs. Stressed/Saline ANOVA *p* < 0.001, Tukey’s test *p* = 0.003; Hypothalamus Stressed/saline vs. Control ANOVA *p* < 0.001, Tukey’s test *p* < 0.001; Midbrain Stressed/saline vs. Control ANOVA *p* = 0.001, Tukey’s test *p* = 0.002; Prefrontal cortex Stressed/Ago vs. Stressed/Saline ANOVA *p* = 0.006, Tukey’s test *p* = 0.005; Nucleus basal ganglia Stressed vs. Control ANOVA *p* < 0.001, Tukey’s test *p* < 0.001, Stressed/Ago vs. Stressed ANOVA *p* < 0.001, Tukey’s test *p* < 0.001.

*PTGS2 (C)* Hippocampus Stressed vs. Control ANOVA *p* = 0.005, Tukey’s test *p* = 0.050; ; Amygdala Stressed/Saline vs. Control ANOVA *p* = 0.001, Tukey’s test *p* = 0.013; Stressed/Ago vs. Stressed/Saline ANOVA *p* = 0.001, Tukey’s test *p* = 0.003; Hypothalamus Stressed/Saline vs. Control ANOVA *p* = 0.010, Tukey’s test *p* = 0.030; Midbrain Stressed/Saline vs. Control ANOVA *p* < 0.001, Tukey’s test *p* < 0.001; Stressed/Ago vs. Stressed/Saline ANOVA *p* < 0.001, Tukey’s test *p* < 0.022; Prefrontal cortex Stressed/Ago vs. Stressed/Saline ANOVA *p* = 0.008, Tukey’s test *p* = 0.01; Nucleus basal ganglia Stressed vs. Control ANOVA *p* < 0.001, Tukey’s test *p* < 0.001.

*IRF1 (D)* Hippocampus Stressed vs. Control ANOVA *p* = 0.003, Tukey’s test *p* = 0.027; Amygdala Stressed/Ago vs. Stressed/Saline ANOVA *p* = 0.003, Tukey’s test *p* = 0.004; Midbrain Stressed/Saline vs. Control ANOVA *p* = 0.003, Tukey’s test *p* = 0.003; Stressed/Ago vs. Stressed/Saline ANOVA *p* < 0.001, Tukey’s test *p* = 0.003; Prefrontal cortex Stressed/Ago vs. Stressed/Saline ANOVA *p* = 0.018, Tukey’s test *p* = 0.017; Nucleus basal ganglia Stressed vs. Control ANOVA *p* < 0.001, Tukey’s test *p* < 0.001.

*IKBKB (E)* Hippocampus Stressed vs. Control ANOVA, *p* = 0.005, Tukey’s test *p* = 0.046; Amygdala Stressed/Saline vs. Control ANOVA *p* = 0.002, Tukey’s test *p* = 0.020; Stressed/Ago vs. Stressed/Saline ANOVA, *p* = 0.002, Tukey’s test *p* = 0.005; Midbrain Stressed/Ago vs. Stressed/Saline ANOVA *p* = 0.015, Tukey’s test *p* = 0.046; Nucleus basal ganglia Stressed vs. Control ANOVA *p* < 0.001, Tukey’s test *p* < 0.001.

### 2.3. Methylation of Studied Genes Promoters

#### 2.3.1. Methylation Status in PBMCs after CMS Procedure and Agomelatine Administration

Neither CMS procedure nor agomelatine treatment resulted in any significant differences in the methylation status of the promoter sequences of the studied *TGFA, TGFB, PTGS2, IRF1* and *IKBKB* genes in PBMCs.

#### 2.3.2. Methylation Status in PBMCs after CMS Procedure and Agomelatine Administration

**Figure 3:**
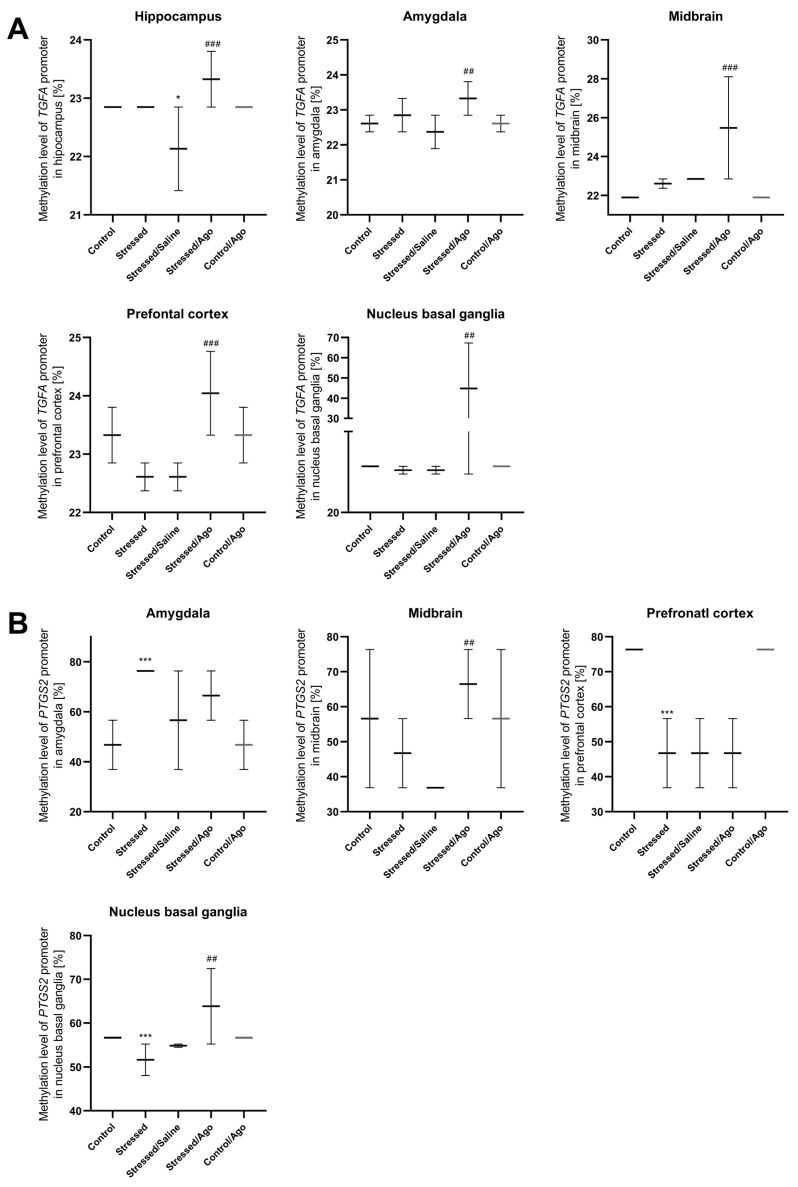

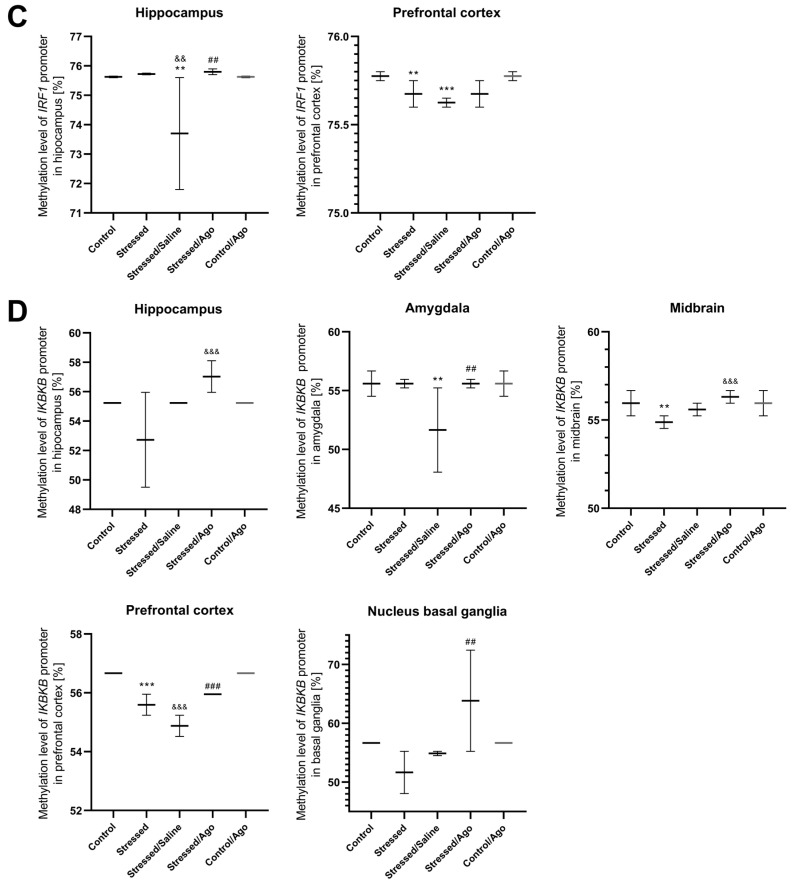
Methylation levels of the *TGFA* (**A**), *PTGS2* (**B**), *IRF1* (**C**) and *IKBKB* (**D**) promoter in brain regions of animals exposed to chronic mild stress (CMS) for two weeks (Control, Stressed) and in animals exposed to CMS for seven weeks including five-week administration of vehicle (1 mL/kg) or venlafaxine (10 mg/kg) (Control/Venla, Stressed/Saline, Stressed/Venla). Data represent means ± SD. *N* = 6. * *p* < 0.05, ** *p* < 0.01, *** *p* < 0.001 relative to Control group. ## *p* < 0.01 ### *p* < 0.001 relative to Stressed/Saline group. && *p* < 0.01; &&& *p* < 0.001 relative to Stressed group.

As shown in Figure 3A–D, the chronic mild stress procedure for 2 weeks caused a significant increase in promoter methylation only in the amygdala of the *PTGS2* gene. However, these animals demonstrated decreased methylation status in the case of *PTGS2, IRF1* and *IKBKB* promoters in their prefrontal cortex as well as the *PTGS2* promoter in the nucleus basal ganglia and *IKBKB* promoter in the midbrain. Interestingly, prolonged seven-week stress induced a decrease in *TGFA* promoter methylation in the hippocampus, *IKBKB* in the amygdala, and *IRF1* in the hippocampus and prefrontal cortex. The chronic administration of agomelatine for five weeks resulted in an increased promoter methylation of *TGFA, PTGS2* and *IKBKB* genes. Specifically, in the case of *TGFA* and *IKBKB*, increased promoter methylation was observed in the hippocampus, amygdala, midbrain, prefrontal cortex and the nucleus basal ganglia. A similar effect occurred in the case of the *PTGS2* gene, where the promoter methylation level was higher in the midbrain and nucleus basal ganglia.

*TGFA* (A) Hippocampus Stressed/Saline vs. Control ANOVA *p* < 0.001, Tukey’s test *p* = 0.027; Stressed/Ago vs Stressed/Saline ANOVA *p* < 0.001, Tukey’s test *p* < 0.01; Amygdala Stressed/Ago vs. Stressed/Saline ANOVA *p* = 0.005, Tukey’s test *p* = 0.003; Midbrain Stressed/Ago vs. Stressed/Saline ANOVA *p* < 0.001, Tukey’s test *p* < 0.001; Prefrontal cortex Stressed/Ago vs. Stressed/Saline ANOVA *p* < 0.001, Tukey’s test *p* < 0.001; Nucleus basal ganglia Stressed/Ago vs. Stressed/Saline ANOVA *p* = 0.002, Tukey’s test *p* = 0.006.

*PTGS2* (B) Amygdala Stressed vs Control ANOVA *p* < 0.001, Tukey’s test *p* = 0.001; Midbrain Stressed/Ago vs. Stressed/Saline ANOVA *p* = 0.014, Tukey’s test *p* = 0.009; Prefrontal cortex Stressed vs. Control ANOVA *p* < 0.001, Tukey’s test *p* < 0.001; Nucleus basal ganglia Stressed vs. Control ANOVA *p* < 0.001, Tukey’s test *p* < 0.001; Stressed/Ago vs. Stressed/Saline ANOVA *p* < 0.001, Tukey’s test *p* < 0.010.

*IRF1* (C) Hippocampus Stressed/Saline vs. Control ANOVA *p* < 0.001, Tukey’s test *p* = 0.005; Stressed/Ago vs. Stressed/Saline ANOVA, *p* = 0.002; Tukey’s test *p* = 0.002; Prefrontal cortex Stressed vs. Control ANOVA *p* < 0.001, Tukey’s test *p* = 0.01; Stressed/Saline vs. Control ANOVA *p* < 0.001, Tukey’s test *p* < 0.001.

*IKBKB* (D) Hippocampus Stressed/Saline vs. Control ANOVA *p* = 0.016, Tukey’s test *p* = 0.010; Stressed/Ago vs. Stressed ANOVA, *p* = 0.001, Tukey’s test *p* = 0.002; Amygdala Stressed/Saline vs. Control ANOVA *p* = 0.016, Tukey’s test *p* = 0.010; Stressed/Ago vs. Stressed/Saline ANOVA *p* = 0.016, Tukey’s test *p* = 0.010; Midbrain Stressed vs. Control ANOVA *p* = 0.001, Tukey’s test *p* = 0.009; Stressed/Ago vs. Stressed ANOVA *p* = 0.002, Tukey’s test *p* < 0.001; Prefrontal cortex Stressed vs. Control ANOVA *p* < 0.001, Tukey’s test *p* < 0.001; Stressed/Ago vs. Stressed/Saline ANOVA *p* < 0.001, Tukey’s test *p* < 0.001; Nucleus basal ganglia Stressed/Ago vs. Stressed/Saline ANOVA *p* < 0.001, Tukey’s test *p* = 0.008.

## 3. Discussion

In the present research, the effect of the chronic mild stress procedure and chronic treatment with agomelatine on the depressive behavior and inflammatory response was explored. We indicated that CMS induced depressive symptoms such as anhedonia, which was measured by the decreased consumption of sucrose solution. Moreover, this effect was accompanied with the dysregulation of inflammatory genes expression. Despite the confirmed involvement of the immune system in depression, knowledge about inflammatory molecules other than cytokines in MDD pathogenesis and its treatment is lacking. To our best knowledge, this is the first study to investigate the impact of agomelatine treatment on the expression of *TGFA, TGFB, PTGS2, IRF1* and *IKBKB* in PBMCs and brain tissues (hippocampus, amygdala, hypothalamus, midbrain, prefrontal cortex and basal ganglia).

Our findings indicate that after the initial two weeks, the mRNA expression of *TGFA, TGFB, IRF1* and *IKBKB* was significantly upregulated in PBMCs. In the case of brain tissues, 2-week CMS caused both an up- and down-regulation of the investigated genes, and results differed between brain structures.

The activation of inflammatory processes in the course of depression could be induced by many factors, and it could be the result of interplay between various biological pathways. One of the proposed causes responsible for the increased expression of variety pro-inflammatory molecules, including cytokines, is disturbances in NF-kB signaling [41,42]. NF-kB activity is regulated, inter alia, by the IKKB kinase encoded by the *IKBKB* gene [38]. There is research confirming the increase in IKKB protein level in the hippocampus after chronic unpredictable mild stress procedure [43]. On the contrary, our findings indicate a diminished expression of the IKBKB gene in the hippocampus after stress stimuli. However, we found that the stress procedure also causes a significant increase in IKBKB mRNA levels in other brain regions, such as the basal ganglia and amygdala. A higher expression of *IKBKB* in PBMCs and some brain regions could contribute to an enhanced activity of NF-kB signaling and thus stimulation of the inflammatory mechanisms during depressive behavior. Interestingly, agomelatine administration reduced CMS-elevated *IKBKB* expression in the amygdala, suggesting anti-inflammatory effects of this antidepressant. Therefore, the proposed method of preventing neuroinflammation accompanying depressive symptoms could be an inhibition of IKKB-mediated NF-kB signaling [43,44].

Another molecule playing an important role in stimulating the production and activity of a number of inflammatory factors is IRF1, which acts as a transcription factor. Furthermore, IRF1 interacts with NF-kB and therefore might be a potential marker of inflammation in psychiatric conditions [35]. In the current research, we have found a higher level of its transcripts after stress stimuli in the nucleus basal ganglia and amygdala. On the other hand, *IRF1* expression in the hippocampus and midbrain was diminished in rats exposed to CMS. The administration of agomelatine was found to reverse the increased *IRF1* expression in the amygdala as well as decreased expression in the midbrain. Based on these results, we could hypothesize that stress might cause a higher expression of *IRF1* and thus increased activity of inflammatory mechanisms but only in some parts of the brain.

Further, TGFB is a pleiotropic factor that stimulates the production of cytokines and other inflammatory agents [45]. Moreover, it is thought to be capable of antagonizing inflammation in the nervous system and exerting a neuroprotective effect in neurodegenerative diseases [15,32]. We have found elevated levels of the *TGFB* gene after 2 weeks of stress procedure in the nucleus basal ganglia and after 7 weeks in the amygdala. These results are consistent with other animal studies indicating its higher expression after chronic unpredictable mild stress [46]. On the other hand, we also observed that *TGFB* levels after the CMS procedure were diminished in the hippocampus, midbrain and hypothalamus. There are also studies that have shown lowered *TGFB* levels in the blood of depressed subjects compared to controls [47,48,49]. Interestingly, the chronic administration of agomelatine normalized the effects of CMS and reduced the stress-elevated levels of *TGFB* in the amygdala. However, treatment with agomelatine also led to an increased expression of *TGFB* in the prefrontal cortex. These and previous data regarding *TGFB*, although inconsistent, show some repeatability. It could be hypothesized that the gene expression may be dependent on brain structures as well as the duration of the stress stimuli. Another investigated member of the TGF family was *TGFA*. We have found that the mRNA levels of the gene were diminished in the midbrain of stressed rats. Simultaneously, *TGFA* expression was significantly up-regulated in the nucleus basal ganglia and amygdala after CMS. In the case of the amygdala, this effect was normalized after agomelatine administration. In addition, long-term stress was manifested by a reduced methylation of the *TGFA* promoter in the hippocampus. What is interesting is that chronic treatment with agomelatine reversed this effect by increasing the methylation status of its promoter in the hippocampus as well as in the amygdala, midbrain, prefrontal cortex and the nucleus basal ganglia. Data regarding role of *TGFA* in psychiatric diseases is lacking; however, it could be advantageous in supporting the neuroprotective effect in the CNS. Specifically, there are reports that TGFA contributes to enhancing the repair mechanism after nervous system post-stroke and post-traumatic injury [50]. Therefore, it might play a beneficial role in maintaining brain plasticity in MDD.

This study also examined *PTGS2,* which is strongly associated with inflammatory processes in the course of depression. Our findings indicate a down-regulation of *PTGS2* expression in the midbrain, hypothalamus and hippocampus, after the CMS procedure. These results are somehow different from those of a previous study indicating an increased *PTGS2* mRNA level in the hippocampus of rats subjected to pharmacological model of depression based on the neonatal administration of clomipramine [51]. Another study also reported higher *PTGS2* expression in rats cortices induced by chronic unpredictable mild stress. [52]. Nevertheless, we have observed an increased expression of PTGS2 in other parts of the brain such as the amygdala and nucleus basal ganglia but not in the hippocampus of stressed rats compared to non-stressed group. In our study, the five-week administration of agomelatine caused increased levels of *PTGS2* in the prefrontal cortex and midbrain. However, agomelatine also induced a down-regulation of *PTGS2* expression in the amygdala, suggesting at least a partial anti-inflammatory action of this drug.

Regarding epigenetics, the CMS procedure and agomelatine administration induced changes in the promoter methylation status. However, promoter methylation modification after stress stimuli was associated with gene expression changes only in the case of *PTGS2* in the nucleus basal ganglia and *IKBKB* in the amygdala. The same applies to agomelatine, although its administration changed the promoter methylation profile; only in the case of *TGFA* was it associated with a change in the expression of this gene in the amygdala. These results could suggest that other factors may have a stronger influence on gene expression regulation than DNA methylation.

It is pertinent to mention that in our study, agomelatine administration was capable of re-establishing sucrose intake to almost a baseline level with at least a fragmental suppression of inflammation in CMS rats. Likewise, previous research studies are in line with this statement. Precisely, agomelatine was found to repress inflammatory signaling and behavioral hallmarks of depression [53]. What is more, agomelatine was shown to modulate the kynurenine pathway, which acts as a key factor in mediating inflammatory-related depression [29]. Moreover, this antidepressant prevented microglial activation and exerted a neuroprotective effect [54,55]. In patients with depressive disorders, agomelatine not only showed anti-inflammatory effects but also increased the expression of BDNF, and hence, it may affect brain plasticity in the pathophysiology of MDD [56].

Our findings mostly support the inflammatory hypothesis of depressive disorders. The CMS procedure caused a characteristic depressive behavior, indicating a significant reduction in the consumption of sucrose solution, which is a generalized deficit in a reward sensitivity accompanied with the dysregulation of inflammatory pathways. Furthermore, these and previous reports allow stating that depression goes along with a variation of immune pathways in the periphery as well as in the central nervous system. Nevertheless, our study had some potential limitations such as the variability of the obtained results between the blood and brain samples as well as among parts of the brain. However, we could speculate as to whether this might be due to a distinct tissue response for stress stimuli or whether other factors may have contributed to these differences. We had used a stable and validated CMS model which closely mirrors depressive behavior; still, results could be biased, since many human symptoms cannot be modeled in animals. Nonetheless, in our opinion, such research brings us closer to the possibility of conducting similar analyses on humans.

In conclusion, the present research supplies evidence suggesting the novel involvement of the previously briefly studied inflammatory genes in the pathogenesis of MDD. Moreover, this study proved that antidepressant agomelatine is able to interfere with factors involved in immune response. It will be therefore important to conduct future research in this direction.

## 4. Materials and Methods

### 4.1. Animals

Male Wistar Han rats, weighing 200–220 g at the start (Charles River Laboratories, Sulzfeld, Germany) were used throughout the experiments. One month before starting the experimental procedures, the animals were brought into the laboratory to acclimate to housing conditions. With the exceptions described below, the rats were housed singly with a maintenance of 12 h light/dark cycle (lights on at 8.00) in controlled room temperature (20 ± 2 °C) and humidity (50% ± 5%). Food and water were allowed ad libitum. All procedures were conducted in compliance with the rules and principles of Directive 86/609/EEC and were approved by the Bioethical Committee at the Institute of Pharmacology, Polish Academy of Sciences, Kraków, Poland (authorization number 1272/2015).

### 4.2. Chronic Mild Stress Procedure

The chronic mild stress procedure was conducted as described previously [57,58]. After accommodating to laboratory and housing conditions, the animals were firstly trained to consume a 1% sucrose solution. The baseline tests were conducted once a week in the home cage. The sucrose solution was presented for one hour after 14 h water and food deprivation. Consumption of the sucrose was monitored once a week, under controlled conditions, until the experiment was ended. Subsequently, on the basis of their sucrose intakes in the final baseline test, the animals were divided into two matched groups. The control group (of non-stressed animals) was housed in separate rooms to exclude contact with the stressed animals. In this group, food and water were freely available except for 14 h deprivation before each weekly sucrose test. The stressed group was exposed to the CMS procedure for a period of two or seven consecutive weeks. Each week of the stress regime consisted of two periods of food and water deprivation, two periods of 45-degree cage tilt, two periods of intermittent illumination (light on and off every two hours), two periods of soiled cage (250 mL water in sawdust bedding), one period of paired housing, two periods of low intensity stroboscopic illumination (150 flashes/min), and three periods without stress. All stressors were applied for 10–14 h and were used individually and continuously, day and night.

### 4.3. Drug Administration

Agomelatine was dissolved in 0.9% sterile saline, which was used for vehicle administration. The drug was then administered IP at a volume of 1 mL/kg of body weight, i.e., a dose of 10 mg/kg, After two weeks of initial stress, the animals were either decapitated or further divided into matched subgroups (*n* = 6) and daily administrated with vehicle (1 mL/kg, IP) or agomelatine (10 mg/kg, IP) for the subsequent five weeks. The drug was administrated to both control and stressed groups. The animals received daily intraperitoneal injections. The weekly sucrose tests were carried out 24 h after the last dose. After the final sucrose test, i.e., after seven weeks of stress, or rather, the completion of five-week administration of vehicle or drug, the animals were decapitated, and blood and brain samples were collected.

### 4.4. Specimen Collection, RNA and DNA Isolation

Peripheral blood samples were collected into 5 mL vacutainers with EDTA. The isolation of peripheral blood mononuclear cells was based on the differential migration of cells during centrifugation. Precisely, blood was mixed with equal volumes of PBS, layered on top of Gradisol L (Aqua-Med) and centrifuged. The interfacial layer (lymphocyte coat) was transferred to a new tube and centrifuged. The supernatant was removed, and PBMCs were stored as pellets at −20 °C until used. After animals decapitation, all brain structures necessary for further experiments were divided and rapidly frozen in liquid nitrogen and stored at −80 °C. Then, a sufficient volume of PBS was added to each sample, which were then homogenized using a FastGene^®^Tisue Grinder (Nippon Genetics Europe, Düren, Germany). The homogenized samples were then sonicated, centrifuged and rinsed with PBS. Prepared samples were used for the isolation of DNA and RNA.

RNA and DNA isolation from PBMCs was performed using the commercial spin column methods with elution in RNAse-Free water (GenElute Mammalian Total RNA Miniprep Kit; Sigma-Aldrich, Saint Louis, MO, USA; QIAamp DNA Mini Kit; Qiagen, Hilden, Germany, respectively) according to the manufacturer’s instructions. RNA and DNA extraction from frozen brain homogenates were conducted with use of the spin column methods by a commercial kit (ISOLATE II RNA/DNA/Protein Kit; Bioline, London, UK) according to the protocol. Total RNA and DNA concentrations were determined spectrophotometrically. The purity of samples was measured as 260/280 nm OD ratio with expected values 1.8–2.0. RNA and DNA samples were stored at −20 °C until further analysis.

### 4.5. Reverse Transcription and Gene mRNA Expression

The reaction of reverse transcription was performed with use of a High-capacity cDNA Reverse Transcription Kit (Biosystems). The total reaction volume was 20 μL. The mixture contained nuclease-free water, 10×RT Buffer, 10×RT Random Primers, 25×dNTP Mix (100 mM), total RNA (0.5 ng/μL) and MultiScribe^®^ Reverse Transcriptase. The reaction tubes were incubated for 10 min at 25 °C, 120 min at 37 °C, and then for 5 min at 85 °C to inactivate the reverse transcriptase. PCR was performed in a C1000™ programmed Thermal Cycler (Bio-Rad Laboratories Inc, Hercules, CA, USA). After the reaction, the cDNA samples were stored at −20 °C. TaqMan Gene Expression Assay was used to examine the expression of the following genes: *IKBKB* (assay ID: Rn00584379_m1), *TGFA* (Assay ID: Rn00446234_m1), *TGFB* (Assay ID: Rn00572010_m1), *IRF1* (Assay ID: Rn01483828_m1), and *PTGS2* (Assay ID: Rn01483828_m1). Reaction was performed using a CFX96™ Real-Time PCR Detection System Thermal Cycler (Bio-Rad Laboratories Inc., Hercules, CA, USA). The housekeeping 18S ribosomal RNA gene (18S) was applied as an internal control (reference gene) for all reverse transcription-quantitative polymerase chains reactions (RT-qPCR). The reaction mixture contained the following: cDNA samples, a TaqMan Universal Master Mix, no UNG (Applied Biosystems, Foster City, CA, USA), TaqMan Probe (ThermoFisher Scientific, Waltham, Massachusetts, USA) and RNAse-free water. The PCR protocol consisted of 10 min at 95 °C (enzyme activation), which was followed by 40 cycles of 30 s at 95 °C (denaturation) and one minute at 60 °C (for annealing/extension). The cycle threshold (Ct) values were calculated automatically by a CFX96 Real-Time PCR Detection System Software System (Bio-Rad Laboratories Inc, Hercules, CA, USA). For each sample, the gene expression of the target mRNA was calculated relative to a reference gene (ΔCt sample = Ct target gene −Ct reference gene). The levels of gene expression are given as a normalization ratio calculated as fold = 2^−ΔCt^ sample.

### 4.6. Methylation-Sensitive High-Resolution Melting (MS-HRM) PCR

The methylation status of investigated gene promoters was evaluated by methylation-sensitive high resolution melting [59,60]. Primers were designed for promoters containing CpG islands using Methyl Primer Express™ Software version 1.0 (TermoFisher Scientific, Waltham, Massachusetts, USA) (Table 2). Firstly, DNA samples (200 ng) were subjected to sodium bisulfite conversion using a CiTi Converter DNA Methylation KiT (A&A Biotechnology), according to the manufacturer’s protocol. PCR was performed using the Bio-Rad CFX96 Real-Time PCR Detection System (BioRad Laboratories Inc., Hercules, CA, USA), and the protocol conditions consisted of 12 min at 95 °C (initial activation), 45 cycles at 95 °C for 15 s, annealing at optimal primer temperatures (tested experimentally) for 20 s, and elongation at 72 °C for 20 s. Methylated DNA (CpGenome™ Rat Methylated Genomic DNA Standard; Merck Millipore) and unmethylated DNA (CpGenome™ Rat Unmethylated Genomic DNA Standard; Merck Millipore) were used as controls for the MS-HRM experiments. To maintain accuracy and control the sensitivity of methylation detection, a series of dilutions had been prepared, namely: non-methylated, 10% methylated, 25% methylated, 50% methylated, 75% methylated and 100% methylated DNA. These reactions were performed using the Bio-Rad CFX96 Real-Time PCR Detection System and analyzed in HRM Powered by Precision Melt Analysis™ Software (Bio-Rad Laboratories Inc., Hercules, CA, USA). Each reaction mixture contained 5 × HOT FIREPol^®^ EvaGreen^®^ HRM Mix (no ROX) (Solis BioDyne), 500 nM of each primer and 10 ng of bisulfite converted DNA (theoretical calculation). The HRM analysis protocol consisted of denaturation at 95 ℃ for 15 s, reannealing at 60 ℃ for one minute and melting from 60 to 95 ℃ at a ramp rate of 0.2 ℃.

### 4.7. Statistical Analysis

The effect of initial two-week stress on sucrose consumption was analyzed by t-test for normally distributed data or the Mann–Whitney rank-sum test for non-normally distributed data. In addition, when the data were normally distributed, the sucrose intake, gene expression and methylation data were analyzed using one-way analysis of variance (one-way ANOVA, SigmaPlot 11.0), with Tukey’s test as a post hoc test; F ratios were significant for the groups Control/Vehicle, Stressed/Vehicle Control/Agomelatine and Stressed/Agomelatine. If the data were not normally distributed, these relationships were tested using the Kruskal–Wallis one-way ANOVA on ranks, which was followed by a post hoc Student–Newman–Keuls test. Student’s *t*-test was used to analyze the differences between blood and brain samples. *p* values < 0.05 were considered significant. Analyses were performed using Statistica 12 (StatSoft, Tulsa, OK, USA), SigmaPlot 11.0 (Systat Software Inc., San Jose, CA, USA) and GraphPad Prism 5.0 (GraphPad Software, Inc., La Jolla, CA, USA).

## 5. Conclusions

In conclusion, the present research supplies evidence suggesting the novel involvement of, unexplored before, inflammatory genes in the pathogenesis of MDD. Our key findings reveal that *TGFA, TGFB, PTGS2, IRF1* and *IKBKB* could be responsible for immune system activation in depression. Specifically, stress stimuli influence the mRNA expression changes of the aforementioned genes in both blood and brain tissues, which successively could trigger an inflammatory cascade. Moreover, this study proved that the antidepressant agomelatine is able to interfere with factors involved in immune response and therefore may demonstrate anti-inflammatory properties. It will be therefore important to conduct future research in this direction.

## Figures and Tables

**Table 1:** Sucrose intakes in animals exposed to chronic mild stress (CMS) for 2 weeks and in animals exposed to CMS for 7 weeks and administered vehicle (1 mL/kg) or agomelatine (10 mg/kg) for 5 weeks.

	Control	Control/ Agomelatine	Stressed	Stressed/ Saline	Stressed/ Agomelatine
**Week 0**	9.85 ± 0.74	12.90 ± 1.83	11.59 ± 1.04	11.30 ±0.89	11.76 ± 0.64
**Week 2**	11.63 ± 1.13	13.27 ± 1.43	5.73 ± 0.98 ***	6.04 ± 1.44 ^###^	6.50 ± 0.72 ^&&&^
**Week 7**		12.46 ± 2.10		5.75 ± 0.44	12.32 ± 1.13 ^@@@^

The data represent means ± SEM. N = 6. *** *p* < 0.001; relative to Week 0 in the Stressed group. ### *p* < 0.001; relative to Week 0 in the Stressed/Saline group. &&& *p* < 0.001; relative to Week 0 in the Stressed/Agomelatine group. @@@ *p* < 0.001; relative to Week 2 in the Stressed/Agomelatine group.

**Table 2:** The specification of primers used for the analysis of methylation levels in the promoter regions of the studied genes.

Gene	Starter Sequence (5′−>3′)	Tm [C°]	Product Size [bp]	Product %CGs
*IKBKB*	F:AGGGTGGTTTTTTATTTTTATTTT R:AACCCCCACTAAAACTAACTTAA	55	117	36.75
*IRF1*	F:TTGGAGATTTAGGGAGTTAGGT R:CCCCTTACCTATCTTAAAAAACC	55	123	43.90
*PTGS2*	F:GTAATAGTAGGGAGGAAAAATTTTAA R:ATCCTAACAAACCCCAAA	55	111	37.84
*TGFA*	F:GTTTTTTTAGGTGGTTGGTTAAG R:CTTCAAACACCTCCCTACAATA	55	188	42.55

## Data Availability

The data that support the findings of this study are available from the corresponding author, [TS], upon responsible request.
